# Leveraging Systems Immunology to Optimize Diagnosis and Treatment of Inborn Errors of Immunity

**DOI:** 10.3389/fsysb.2022.910243

**Published:** 2022-07-18

**Authors:** Andrea A. Mauracher, Sarah E. Henrickson

**Affiliations:** 1 Division of Allergy and Immunology, Department of Pediatrics, Children’s Hospital of Philadelphia, Perelman School of Medicine, University of Pennsylvania, Philadelphia, PA, United States,; 2 Department of Microbiology, Perelman School of Medicine, University of Pennsylvania, Philadelphia, PA, United States,; 3 Institute for Immunology, Perelman School of Medicine, University of Pennsylvania, Philadelphia, PA, United States

**Keywords:** inborn errors of immunity, STAT1 GOF, primary immunodeficiencies, systems immunology, multimodal data analysis

## Abstract

Inborn errors of immunity (IEI) are monogenic disorders that can cause diverse symptoms, including recurrent infections, autoimmunity and malignancy. While many factors have contributed, the increased availability of next-generation sequencing has been central in the remarkable increase in identification of novel monogenic IEI over the past years. Throughout this phase of disease discovery, it has also become evident that a given gene variant does not always yield a consistent phenotype, while variants in seemingly disparate genes can lead to similar clinical presentations. Thus, it is increasingly clear that the clinical phenotype of an IEI patient is not defined by genetics alone, but is also impacted by a myriad of factors. Accordingly, we need methods to amplify our current diagnostic algorithms to better understand mechanisms underlying the variability in our patients and to optimize treatment. In this review, we will explore how systems immunology can contribute to optimizing both diagnosis and treatment of IEI patients by focusing on identifying and quantifying key dysregulated pathways. To improve mechanistic understanding in IEI we must deeply evaluate our rare IEI patients using multimodal strategies, allowing both the quantification of altered immune cell subsets and their functional evaluation. By studying representative controls and patients, we can identify causative pathways underlying immune cell dysfunction and move towards functional diagnosis. Attaining this deeper understanding of IEI will require a stepwise strategy. First, we need to broadly apply these methods to IEI patients to identify patterns of dysfunction. Next, using multimodal data analysis, we can identify key dysregulated pathways. Then, we must develop a core group of simple, effective functional tests that target those pathways to increase efficiency of initial diagnostic investigations, provide evidence for therapeutic selection and contribute to the mechanistic evaluation of genetic results. This core group of simple, effective functional tests, targeting key pathways, can then be equitably provided to our rare patients. Systems biology is thus poised to reframe IEI diagnosis and therapy, fostering research today that will provide streamlined diagnosis and treatment choices for our rare and complex patients in the future, as well as providing a better understanding of basic immunology.

## 1 INTRODUCTION

### 1.1 Historic Perspective

While concept of health and disease is as old as our species, the concept of measuring the health of the immune system is comparably recent and still developing today. The field of immunology arguably emerged out of our desire to understand and treat infectious diseases (Ochs and Hitzig, 2012) and as such the concept of a healthy immune system has been the absence of infections or simply the ability to live a long life. In the late 19th and early 20th century, we began to understand that the immune system could be therapeutically modulated by vaccinations and supported by antibiotics. In turn, the burden of infectious diseases rapidly decreased. It was during this period that various attentive physicians recognized that groups of patients, susceptible to specific patterns of infections despite treatment, had deficiencies in key components of the immune system (Notarangelo and Casanova, 2009; Ochs and Hitzig, 2012). Over the following years, the number of recognized primary immunodeficiencies increased, each with specific inheritance patterns and, initially, almost all identified based on increased infectious susceptibility (Seligmann et al., 1968).

As the number of recognized primary immunodeficiencies (PIDs) increased, the need for a classification system arose, leading to the first PID classification in 1968 (Seligmann et al., 1968; Fudenberg et al., 1970). This initial classification was largely based on the “suggested cellular defect.” Almost simultaneous to this initial classification Adenosine deaminase deficiency (ADA) deficiency was discovered to be the first PID associated with a genetic defect (Ochs and Hitzig, 2012). This start of the genetic age in clinical immunology would soon fundamentally alter how we classify PIDs, with a focus on attempting to link every disease to a disease-causing gene(s). Over the following years, the field naturally expanded in parallel with the use of genetic testing. This is evident in the almost exponential increase we have seen in genetically defined PIDs in the past decade (Bousfiha et al., 2020; Notarangelo et al., 2020; Tangye et al., 2020; Tangye, 2021a; Tangye, 2021b). This increase was potentiated by our expanding understanding of the symptoms that define a PID. The field noted that in some PIDs, autoimmunity, lymphoproliferation and malignancies were quite common, sometimes even pathognomonic. These, and other symptoms, as an expression of immune dysregulation, define a subset of PIDs better than simply an increase in infectious susceptibility (Fischer et al., 2017; Mauracher et al., 2021). In fact, the number of diseases that meet these criteria, termed “primary immune regulatory disorders (PIRD)”, have rapidly increased over the past 5–10 years (Chan and Torgerson, 2020). This shift is evident in the implementation of the term “Inborn Errors of Immunity (IEI)” (versus PID) to include these patients in the most recent IEI classification (Notarangelo et al., 2020).

#### 1.1.1 Current Challenges in Diagnosing and Treating Inborn Errors of Immunity

The current International Union of Immunological Societies (IUIS) classification of IEIs is based on clinical phenotype and clinical testing, including defining immune phenotype and function and gene sequencing (Bousfiha et al., 2020). This classification in many ways reflects the various aspects required to diagnose an IEI patient. The implementation of T-cell receptor excision circle (TREC)-based Severe Combined Immunodeficiency (SCID) newborn screening across the United States and building around the world has accelerated the diagnosis of IEI patients with both SCID and other causes of T cell lymphopenia. This advance permits identification and treatment of these patients before the first symptoms begin (Mauracher et al., 2017; Buchbinder et al., 2021; Currier and Puck, 2021). Nonetheless, the majority of IEI patients across the world still initially present to clinical immunologist years into persistent, complex symptoms that are suggestive of an IEI. Thus, clinical astuteness is required to recognize the warning signs of IEI. Thus, clinical astuteness is required to recognize the warning signs of IEI. The extensive clinical workup of a patient is central in establishing the suspicion as well as the diagnosis of an IEI. Similarly, laboratory parameters are important initial investigations that nearly all patients receive. These usually include the quantification of serum immune factors and immune cell subset frequency and function. Depending on the initial suspicion, the laboratory evaluation of a patient can become specific, if not even diagnostic. Finally, if an IEI is suspected, genetic sequencing is usually additionally performed. This can entail anything from Sanger sequencing of a specific gene known to be epidemiologically prevalent to Whole Genome Sequencing (WGS) in centers experienced herein. The specifics of each of these aspects to diagnosis as well as timepoints they are applied can vary substantially depending on the epidemiological background of a country, the experience of the clinical team and the tests and expertise available.

Along this diagnostic odyssey, with best intentions, patients are often misdiagnosed, unclear results are common and issues around the accessibility to the diagnostic tests required are still extensive (Meyts et al., 2020a). Thus, it is not surprising that diagnostic and therapeutic delays are still common for IEI patients and result in an increased morbidity and reduced quality of life (King et al., 2021; Anderson et al., 2022). There are many ways by which these diagnostic delays have effectively been addressed over the past years. Here, we must undoubtably note the remarkable advances the increased accessibility in next generation sequencing (NGS) methods, specifically gene panels, whole exome sequencing (WES) and WGS have brought. These technologies have increased our understanding of IEI to the point, that genetics has justifiably become the gold standard of diagnosing IEI (Tangye et al., 2020; Tangye, 2021a). Nonetheless, there are still many patients for whom genetic sequencing does not yield conclusive results. In patients where neither laboratory tests nor genetics are conclusive, we are limited to basic research based functional evaluations of interesting patients. Furthermore, as a field we still grapple with how to deal with novel variants in known disease-causing genes (e.g., “variants of unknown significance”; VUS) or novel potential disease-causing genes (e.g., genes with links in animal data to the phenotype seen in the patient) (Meyts et al., 2016a; Meyts et al., 2020a). In these situations, the impact of these variants on disease needs to be systematically assessed, which can be time consuming or even unrealistic (Casanova et al., 2014a). Finally, even when a genetic diagnosis is available there are increasing variants that cannot fully inform treatment, this is especially true for PIRDs.

#### 1.1.2 Addressing These Challenges

Thus, as we as a field have transitioned to an earlier focus on genetic testing, we now need to optimize how we proceed past our genetic findings, when necessary, while remaining informed by them. This requires us to optimize our mechanistic studies of protein and cellular function evaluating whether a genetic variant together with the individual immune state yields a functional outcome that can impact therapeutic choices (Levy et al., 2017; Taylor et al., 2019; Akdis, 2021; Hay and Henrickson, 2021; Porsche et al., 2021). We can do this by using evidence-based high-dimensional strategies to identify key pathways and then developing and validating more robust, targeted functional tests based on the identified key pathways. These results can allow us to expand our diagnostic algorithm to facilitate the identification of core dysregulated signaling pathways in IEIs. This information, in adjunct with genetics, can allow us to functionally diagnose our patients and facilitate targeted, personalized treatments.

In this review we will lead readers through the current methods that are being used to diagnose and study IEI patients and how related systems biology methods are beginning to change how we study and understand IEI. We will expand on what endeavors are needed to effectively integrate systems biology approaches into our clinical immune testing in the future. Finally, we make the point that when thoughtfully and collaboratively established, systems biology can provide a pathway- and function-based approach to classifying IEI. This could in turn provide a basis to quickly identify and flexibly validate personalized targeted therapies.

## 2 DEFINING HEALTHY VARIABILITY

**FIGURE 1 |:**
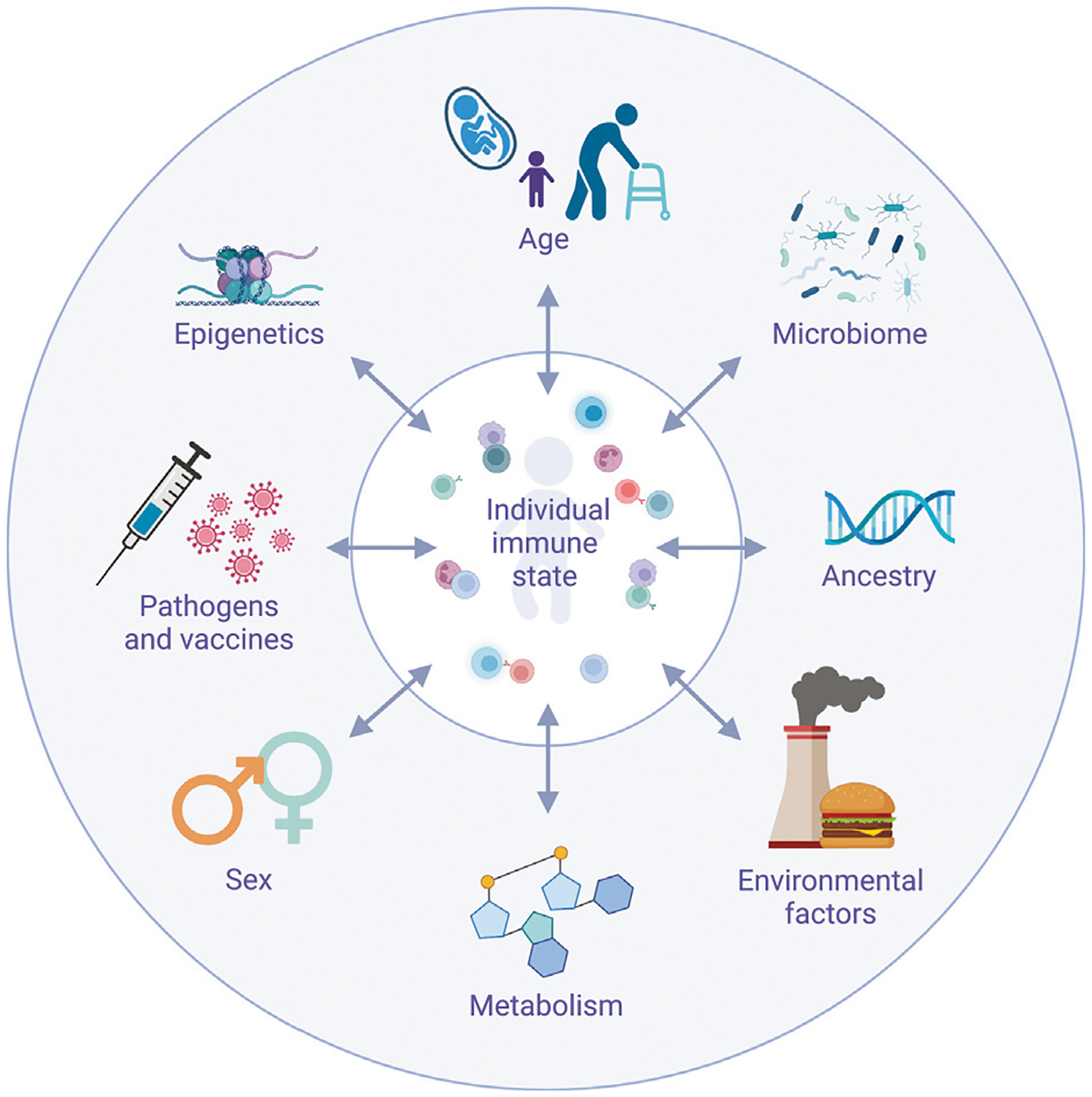
Factors influencing immune cell subset frequency and function.

Before we introduce the various methods used to diagnose IEI patients, we will briefly review some of the many factors that modulate a healthy immune system (Figure 1) (Levy et al., 2017; Duffy, 2018; Taylor et al., 2019; Akdis, 2021; Hay and Henrickson, 2021; Porsche et al., 2021).

### 2.1 Why Do We Need to Quantify Healthy Variability?

**FIGURE 2 |:**
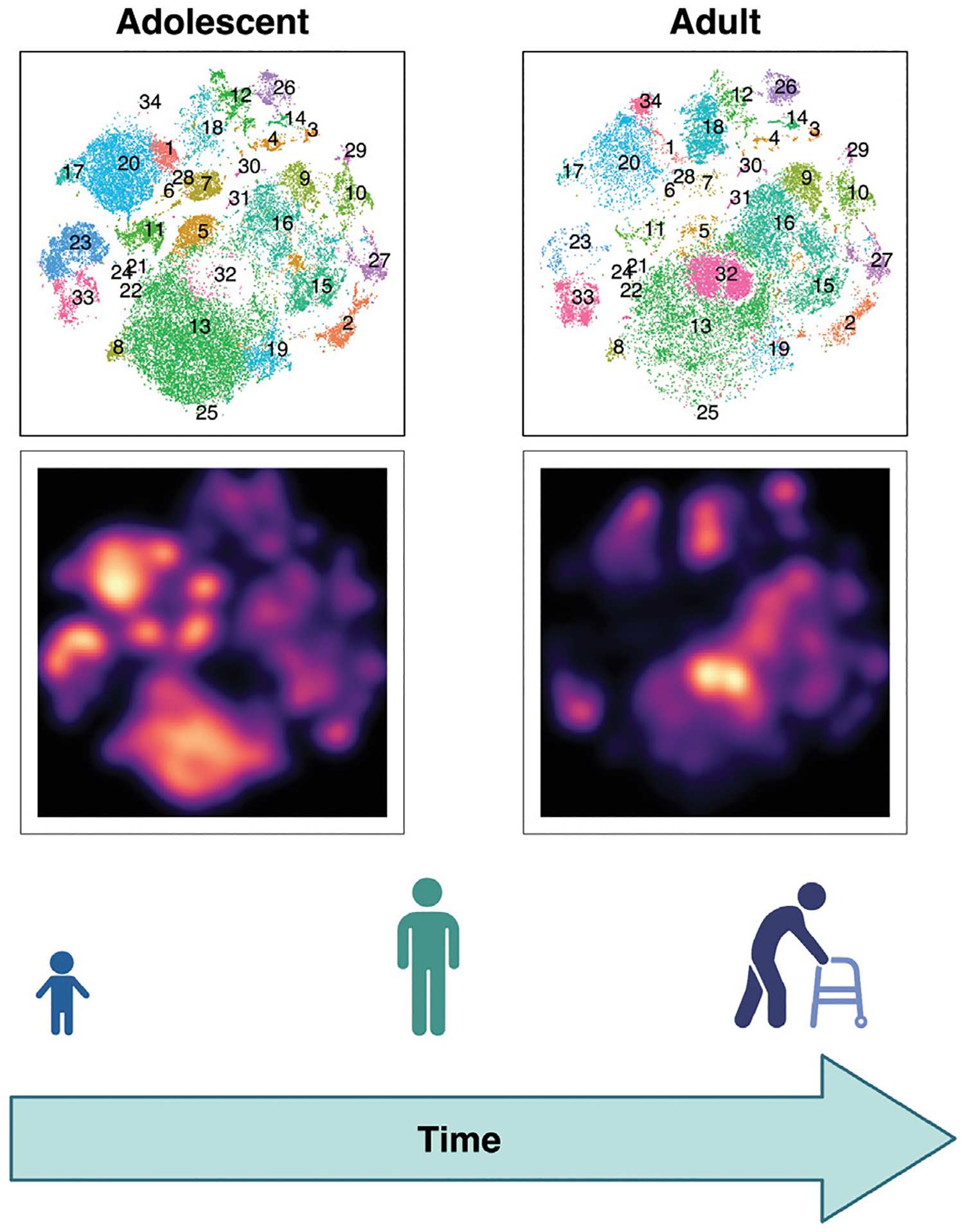
Evolution of T cell subset balance over lifespan visualized with high dimensional immune profiling. Peripheral blood mononuclear cells (PBMCs) were stained using metal conjugated antibodies and evaluated via CyTOF. T cells from adolescent (left) versus adult (right) healthy participants are compared. Map of T cell clusters (above) and galaxy plots (below) demonstrate the differences between age groups.

When studying disease, healthy controls have long been seen as individuals who do not have the condition and, in the past, have often been treated as a relatively homogenous group. While this approach is not always wrong, it can lead to false conclusions. At the most basic level, we know that children have different proportions and differentiation states of immune cells, which vary with age (Taylor et al., 2019; Shearer et al., 2003; Garcia-Prat et al., 2019; Fulop et al., 2018; Lakshmikanth et al., 2020; Olin et al., 2018; Alpert et al., 2019) (Figure 2). Thus, using adults (e.g., their parents or older siblings) as controls for pediatric patients with IEIs could lead one to falsely conclude that an adequate immune cell composition for the patient’s age is abnormal and thus disease relevant. While age is a key factor to consider when selecting controls, there are many potential confounders we might need to account for, including CMV status. Given our current limited understanding of the demographic factors that affect immune function, we may be missing other key factors. Therefore, as a field we need to broadly study healthy interindividual variation to evaluate factors that impact immune cell subset balance and function (Figure 1) (Brodin et al., 2015; Duffy, 2018). This means we need to ensure our healthy cohorts are diverse and representative by including individuals across sex, age, ancestries, socioeconomic levels, geographic locations and more (Rozenblatt-Rosen et al., 2017; Taylor et al., 2019; Haniffa, 2021; Sayed et al., 2021).

The need to better define human heterogeneity is becoming clearer as systems biology approaches are allowing us to more deeply assess each individual’s immune phenotype. Many of these emerging methods can capture an increasing number of parameters, leading to a growing level of data complexity. This complexity can reveal heterogeneity in “healthy individuals” formerly not recognized (Figure 2) (Brodin et al., 2015). Currently, we need to define reference ranges for each technique we choose to use, or at least include a significant pool of healthy controls in our research, carefully considering the needed axes of variation among them. Only once we do this, can optimally use these methods to uncover relevant disease mechanisms.

### 2.2 How Do We Define Healthy Variability?

To grasp the full breadth of healthy immune variability, one challenge is how to feasibly include diverse, healthy individuals as equitably as possible; how do we ensure that our selection of healthy controls is representative and not biased by external cofounders. In the field of genetics, the importance of establishing diverse, representative healthy cohorts in increasing our understanding of disease is well documented (Abul-Husn et al., 2019; Abul-Husn and Kenny, 2019). Today, we know that each individual’s genome contains millions of single nucleotide polymorphisms (SNPs). This healthy variation in part constitutes an individual’s genetic background and this clear understanding of the range of variation in the healthy populations has been a prerequisite to identifying disease-causing variants. Specifically, to assess the potential relevance of a novel disease-causing variant, we use large, publicly available datasets to confirm that the variant is not present in a significant proportion of the healthy population. It is in these databases where the effects of bias have been well documented (Abul-Husn and Kenny, 2019). When these databases were initially constructed, they largely included genetic data on individuals of European descent (Need and Goldstein, 2009; Altman et al., 2016). This limits their practicality for diagnosis and research on large subsets of the world population and is a pressing issue that is currently being addressed and is beyond the scope of this review (Abul-Husn et al., 2019; Abul-Husn and Kenny, 2019; Belbin et al., 2021).

This public data nonetheless constitutes one of the largest efforts to capture healthy heterogeneity to date and teaches us important lessons on the importance of unbiased inclusivity. For many emerging systems biology techniques, efforts are currently underway to create similar publicly available databases (e.g., Human Cell Atlas for single cell transcriptomics). As part of this, challenging discussions around the thoughtful and inclusive selection of patients are present and efforts underway (Rozenblatt-Rosen et al., 2017). The ongoing Pediatric Cell Atlas is such an example, aimed at expanding the human cell atlas to also account for the relevant cellular variation with age (Taylor et al., 2019). Concerted efforts are now required, to ensure that these databases are created with a focus on equity and inclusion, to represent the healthy individuals of all genders, ethnicities, geographical locations, ages, socioeconomic backgrounds and more. As part of this effort to equitably evaluate healthy heterogeneity, one question that inevitably will arise is, where do we draw the line between health and disease?

### 2.3 Where Does Healthy Variability End and Immune Dysfunction Begin?

Defining the bounds of normal immune variation is challenging (Brodin and Davis, 2017; Yan et al., 2021). The immune system is a highly complex and connected system, in which immune cells interact with and react to each other but also to non-immunologic cells, microbes, environmental influences and metabolites (Figure 1) (Duffy, 2018). Therefore, a healthy immune system is a dynamic system that is constantly altering its composition and function in response to myriad perturbations (Taylor et al., 2019). While some of these changes are adaptive and support immune function, we also need to be able to recognize at what point they are pathologic and become clinically relevant.

Furthermore, throughout our lives our immune systems are in contact with the microbes in our environment, including those that are environmental, commensal, and pathogenic (Duffy, 2018). While our commensal microbes educate our immune systems and contribute to proper immune cell function, pathogens and inflammation also lead to lasting alterations. Viruses including as Epstein-Barr virus (EBV) or Cytomegalovirus (CMV) are chronic infections that substantially alter our immune composition (Brodin et al., 2015; Alpert et al., 2019; Yan et al., 2021). As we study healthy immune cell variation, in the future we will need to account for how infections and inflammation acutely and chronically affect the immune system and define how to properly control for this, when analyzing results from patients with or without the corresponding infections. Properly assessing for this interplay that naturally occurs between our immune system and pathogens is challenging, as infections may not always be clinically recorded (especially if only associated with mild symptoms) in healthy individuals. Nonetheless, the study of this interplay will be paramount in delineating what constitutes a healthy immune response vs. pathological inflammation.

There are many known factors that contribute to the variation we see in the composition and function of healthy immune systems and many unknown factors are yet to be discovered. In the next sections we will focus on some of the systems biology-based technologies currently being used to understand and assess the composition and function of the immune system.

## 3 TECHNIQUES

### 3.1 Genetics

IEI are currently classified by the IUIS (Tangye et al., 2020). This classification system categorizes patients based on laboratory findings, clinical phenotype and genetic variant (when known). For example, in the category of “Immunodeficiencies affecting cellular and humoral immunity” the various forms of Severe Combined Immunodeficiency SCID (T-B+ or T-B-SCID) or combined immunodeficiency (CID) can be found together with the genetic entities that can underlie these diseases. Based on this classification system, genetics is considered the gold-standard for diagnosing a patient with a suspected IEI (Tangye et al., 2020). As this is not always feasible, for various reasons, in clinical practice patients are often treated based on clinical phenotype and laboratory-based diagnosis. This is important, as therapy should not be withheld if a genetic diagnosis is not known. Nonetheless, genetics can provide essential information, including optimization of therapeutic selection, and is therefore justifiably central in IEI diagnostics. which presents similarly to a subset of Severe Combined Immunodeficiency (SCID), but secondary to congenital athymia, or DNA repair defects presenting as SCID We will next discuss how genetics are currently applied in IEI diagnostics and where the challenges lie.

#### 3.1.1 Genetic Testing Strategies

Although advances in NGS technologies, such as IEI gene panels, WES and WGS have allowed us to diagnose increasing numbers of patients, access to these technologies is still an issue worldwide (Heimall et al., 2018a; Meyts et al., 2020a; Meyts et al., 2020b; Karimi et al., 2021a; Engelbrecht et al., 2021). While smaller hospitals or centers located in low-income regions may not have access to NGS techniques, or only smaller scale strategies such as gene panels, larger centers may also only have access to these on a research basis or for some insurance options there may be reduced access to many or all these strategies (Rudilla et al., 2019; Villavicencio and Pedroza, 2019; Engelbrecht et al., 2021; DInur-Schejter and Stepensky, 2022). In places throughout the world where only Sanger sequencing for selected variants or limited gene panels are available, often only selected genes can be assessed for possible IEI patients. Here clinicians, guided by the patient phenotype and the available laboratory parameters, will decide which genes, or even what specific variant to test for (Heimall et al., 2018b; Karimi et al., 2021b). Even for the most experienced clinicians, this is extremely challenging and is best addressed by increasing access to broad NGS.

Increasing access to NGS over the last years has highlighted the impressive phenotypic overlap in patients presenting with variants in different genes or phenotypic breadth of patients with variants in the same gene (Notarangelo et al., 2020; Mauracher et al., 2021; Chan et al., 2020; el Hawary et al., 2019). Similarly, incomplete genetic penetrance, which we are increasingly recognizing to be common in IEI, can makes inheritance patterns difficult to recognize based on family history (Seleman et al., 2017; Olbrich and Freeman, 2018; Notarangelo et al., 2020). Finally, mosaicism or somatic mutations, increasingly shown to be relevant contributors to IEI, can lead to unique clinical presentations that can be technically difficult to discover genetically, though this is evolving (Bousfiha et al., 2020; Lee et al., 2020; Aluri et al., 2021).

#### 3.1.2 Limitations of Whole Exome Sequencing and Whole Genome Sequencing

Generating and analyzing WES or WGS data requires an experienced team of bioinformaticians, clinicians and researchers alike to establish flexible pipelines (Meyts et al., 2016a; Seleman et al., 2017). As part of the analysis of large genetic datasets, defined filtering and ranking algorithms are usually applied to reduce the list of candidate variants. Therefore, when viewing results, clinicians ideally should be aware of how for their specific datasets and analysis pipelines, relevant variants might be lost. For example, there can be an impact on results based on how the clinical phenotype is communicated to the analysis team. Without clear description of the immune alterations (most often using Human Phenotype Ontogeny, or HPO, terms (Köhler, 2019)) algorithms can filter out genes that are currently not understood to involve in the immune system. Similarly, ranking potential variants, based on their predicted effect on the resulting protein, can falsely prioritize variants as foretelling the effects of a variant on gene function is difficult.

Variants in the same gene, in fact sometimes of the same nucleotide, can have widely diverging effects on a protein level and/or function, leading to a gain- or loss-of-function (GOF or LOF) variants, both of which can be relevant (Picard and Fischer, 2014; Chandrasekaran et al., 2016; Heimall et al., 2018a). Therefore, pipelines that prioritize autosomal recessive variants, which are usually also LOF variants, can introduce a false bias (Pedersen et al., 2021). Furthermore, current algorithms are optimized to recognize monogenic germline variants, not polygeneic diseases or somatic mutations (Pritchard, 2001; Casanova and Abel, 2005; Silva et al., 2019; Aluri et al., 2021). There are also some pitfalls inherent to the chosen methodology. Gene panels and WES are often optimized for exonic variants and tend to miss intronic variants or large deletions (Meyts et al., 2016b; Heimall et al., 2018b). Furthermore, in WES the library preparation beforehand, and the baits used for exome enrichment, can both influence coverage of genes (Picard and Fischer, 2014; Meyts et al., 2016b; Heimall et al., 2018b) and finally, WES can miss copy number variants and analysis of genes that contain pseudogenes can be complex (Heimall et al., 2018b). Overall, with current WES strategies and well-selected patient cohorts, we expect to find causative variants in ~10%–40% of patients (Yang, 2013; Maffucci et al., 2016).

The above is by no means an exhaustive account of all the potential contributors to the fact, that for a substantial proportion of IEI patients a genetic diagnosis cannot be found, including the potential that there may simply not be one for any given patient. As we increase access to NGS, and become more proficient in its analysis, it is fair to expect many more genetic entities will arise in the future and we will more quickly recognize disease-causing variants (Itan and Casanova, 2015). Nonetheless, we will continue to require validation of VUS in a known genes or novel genes on a case-by-case basis—which is a challenge (Casanova et al., 2014b; Picard and Fischer, 2014; Meyts et al., 2016b). This time-consuming process will most likely continue to lead to further diagnostic delays. When we additionally consider the aforementioned phenotypic variability present in patients, that cannot be explained by genetics alone, and the fact that a genetic variant in many cases cannot inform therapy, we need to ask ourselves whether this purely genetics-based approach to IEI is sufficient. This is where adjunctive methods to diagnose patients come in.

### 3.2 Immune Profiling

#### 3.2.1 Phenotyping Immune Cells During Clinical Evaluation

When the first PID classification grouped known diseases based on affected cell type, the methods used to differentiate immune cells were still very complex and time consuming (Ma and Tangye, 2019). Flow cytometry has revolutionized this process by allowing for the quick and accurate characterization and enumeration of immune cells (Herzenberg and Herzenberg, 2004). Today, flow cytometry is essential in the diagnostic workup of patients with suspected IEI. Enumerating B-, T- and NK-cell frequency, a process that once was time consuming, is now simple and generally accessible, and can quickly point clinicians towards an IEI that includes absence or dramatic reduction of an immune cell subset (Buckley, 2004; Bousfiha et al., 2020). This basic assessment is crucial in the rapid evaluation for SCID and provides a basis by which to categorize patients and decide on further diagnostic steps (Madkaikar et al., 2019; Rawat et al., 2019). Over the past years, parameters that can be analyzed by basic flow cytometry have increased, allowing increasingly detailed immune phenotyping as part of clinical evaluation, providing more specific information on IEI patients. However, the use of this increased depth of immune profiling in an evidence-based fashion is yet somewhat limited, as prognostic information has not been correlated or connected to clinical metadata in many cases. Nonetheless, these advances have in some disorders allowed flow cytometry to the capacity of providing a near definitive indication as to a patient’s genetic diagnosis (Kroczek, 1994; Futatani et al., 1998; Grimbacher et al., 2003; Palendira et al., 2012; Pai et al., 2014; Cabral-Marques et al., 2019; Ma and Tangye, 2019). An example of this is X-linked agammaglobulinemia, where it can be diagnostic when a patient is found to lack B cells and have absent Bruton’s tyrosine kinase (Btk) *via* flow cytometry (Futatani et al., 1998).

#### 3.2.2 Phenotyping Immune Cells in Research

While some advances in traditional flow cytometry have reached clinics, techniques such as high dimensional immune cell profiling techniques such as Cytometry by Time of Flight (CyTOF) are still largely bench-based. CyTOF has substantially increased the scale of parameters that can be simultaneously analyzed in a given sample (Saeys et al., 2016; O’Boyle, 2020). Thus, rare immune cell populations can be identified without requiring an increased sample volume, a specific advantage when working with IEI patients, who are often children and may be lymphopenic. Although CyTOF has transformed immune profiling, it has its disadvantages, including low rate of cell analysis, batch effect between runs and between batches of antibody conjugation, and cost. Emerging cytometry technologies, including spectral flow cytometry, are cheaper, generally make use of fluorophores already established in diagnostic laboratories, while promising to allow for a similar, though somewhat reduced, number of parameters to be analyzed (Saeys et al., 2016). This has resulted in the recognition of novel, rare disease-associated immune cell subsets defined by combinations of intra- and extracellular markers and subsequent research into function and role of these rare populations in health and disease. This research provides the basis to recognize relevant markers to be integrated into clinical panels in the future. Given the complexity of working with heavy metal labeled antibodies and the highly specialized nature of the technique itself, it may be less likely that CyTOF would become part of a clinical pipeline and spectral flow cytometry may be more likely. Either way, discoveries made using these techniques will inform clinical flow cytometry moving forward regardless of the technique used in that setting.

There are various examples of how these high-dimensional immune profiling techniques can be used to increase our understanding of differences and similarities between patient cohorts. In recent reports CyTOF has been used to identify differences in the immune cell profiles of patients with Multisystem Inflammatory Syndrome in Children (MIS-C) vs. Coronavirus disease 19 (COVID-19) and healthy controls, substantially contributing to our understanding of the differing disease pathogenesis in these cohorts (Consiglio et al., 2020; Ramaswamy et al., 2021; Vella et al., 2021). Another recent publication used CyTOF to identify patterns associated with early the immune aging in a cohort of Down Syndrome patients as a potential mechanism underlying their autoimmunity (Katharina, 2022). This exemplifies how CyTOF, and high dimensional immune profiling more generally, can be used to gain important insights into patient cohorts by initially studying healthy variation. First applications of high dimensional immune profiling in IEI patients are still rare, but it has been used as an approach to distinguish CVID patients where a genetic diagnosis is not available (Emmaneel et al., 2019; Silva et al., 2019).

It is important to note that these techniques can be used for both immune profiling and functional evaluation. While phenotypic insights into of patient cohorts can suggest mechanistic differences, functional evaluation is central in illuminating the pathways involved in this dysfunction. The increased dimensionality that can be assessed in one sample via these strategies, compared to traditional flow cytometry, also opens up possibilities for broad functional assessment in parallel with this phenotypical characterization.

#### 3.2.3 Functional Testing

**FIGURE 3 |:**
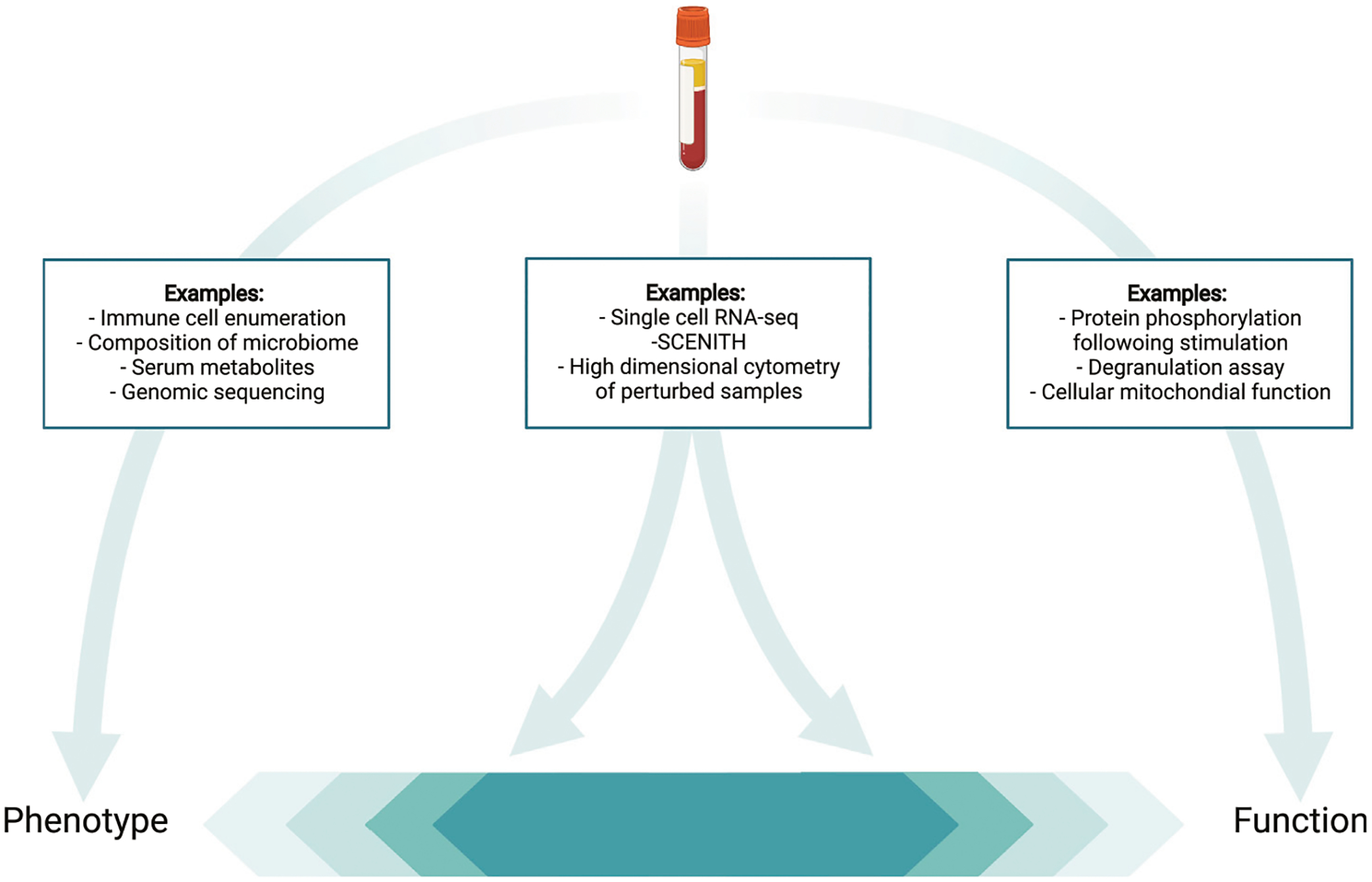
The challenge of defining techniques as phenotypic vs. functional. Depicted are examples of techniques that provide purely phenotypical data (left) or largely functional data (right) in contrast with approaches that can provide both functional and phenotypic insights (middle).

Functional insights into immune cell subsets can be achieved by combining specific antibodies for cell subset identification with those against target proteins such as cytokines or signaling proteins that measure effector function, impacted by targeted perturbations (e.g., stimulation with cytokines). Classic examples of functional evaluation via flow cytometry include degranulation assays for hemophagocytic lymphohistiocytosis (HLH) or the detection of dihydrorhodamine as a test for Chronic Granulomatous Disease. Using classic flow cytometry, functional assays are often conducted separate from phenotyping. In part, limited parameters available per analysis hinder the two from being combined (Figure 3). This in turn means that functional evaluation requires additional blood volume and separate approval, potentially making this more difficult. High-dimensional profiling theoretically addresses this issue, by allowing functional assays to be combined with detailed immune cell phenotyping providing mechanistic insights in parallel with phenotypic detail. However, it is important to note that approval of a clinical test requires extensive validation, and this important quality control work may complicate combination of multiple tests.

**FIGURE 4 |:**
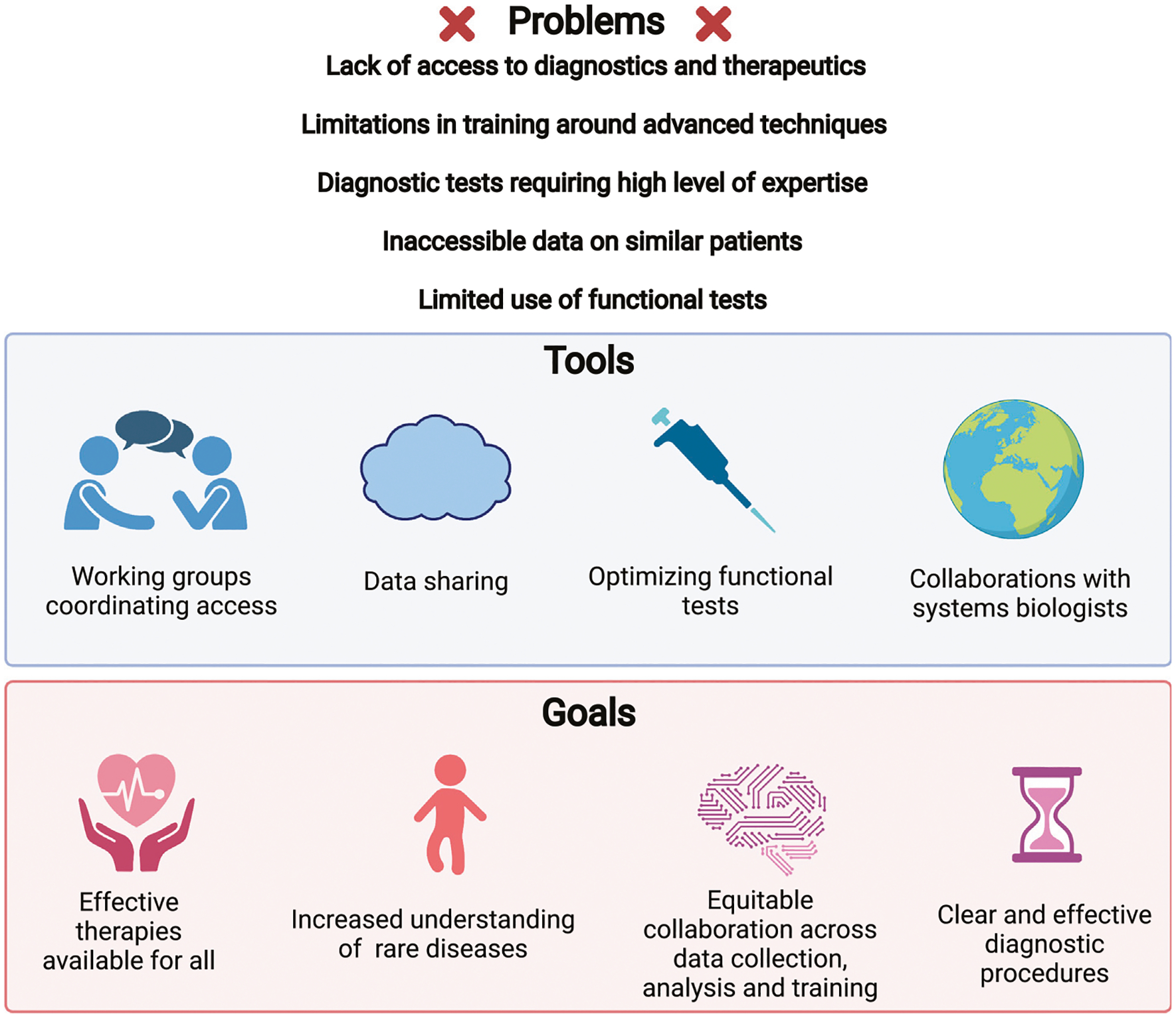
Challenges leading to diagnostic delays and impeding research into IEIs. Problems that have been noted in the clinical and research study of IEIs (upper). Strategies being implemented to address these issues (middle). Goals for our field, potentially achievable using these strategies (lower).

Currently, standardized functional testing conducted as a part of immune profiling is still very limited. Many specific functional tests are not available in resource poor settings, and most are only possible in the context of basic research. It remains to be seen if high dimensional immune profiling can identify effective functional tests to be optimized and to provide actionable results and made more accessible. For the time being, collaborative efforts aimed at providing access to functional testing may best bridge this gap (Figure 4).

### 3.3 Transcriptomics

Beyond germline genetic variants, NGS can provide insight to assess the level of gene expression in a cell type of interest, *via* analyzing RNA levels. When the transcription of a single or a few genes are being evaluated, quantitative PCR (qPCR) is an ideal strategy. Transcriptomics on a broader scale, also known as RNA sequencing (RNA-seq), can provide a qualitative and quantitative overview of all the coding and non-coding RNAs transcribed within a cell (single cell RNA-seq; scRNA-seq) or a cellular population (bulk RNA-seq). The former allows evaluation of the heterogeneity in gene expression between cells that share a single cell subset. Finally, microarray based sequencing technologies (e.g., NanoString), present methods allowing the targeted quantification of transcriptional signatures. While all of these are powerful tools, their applications in IEI remain limited and when, are mostly conducted on bulk cells.

With regards to diagnostics, when variants are identified in WES or WGS in genes known to be expressed in hematopoietic cells, RNA-seq can be used to assess the impact of potential IEI causing variants on gene expression (van Schouwenburg et al., 2015; Khan et al., 2016). For variants in genes that act as transcription factors, such as Signal Transducer and Activator of Transcription (STAT), RNA-seq can provide information on whether a suspected variant causes a LOF or GOF in protein activity, resulting in downstream transcriptional alterations (Mauracher et al., 2020). In patients with common variable immune deficiency (CVID), where disease-causing variants are found in a minority of patients, whole blood RNA-seq has been used to identify patients in whom an upregulation in interferon (IFN) responsive genes (ISGs) was linked to an increased risk of inflammatory complications (Park, 2013; Ho and Cunningham-Rundles, 2020). This suggests that whole blood RNA seq could provide a means to categorize a subset CVID patients at risk for inflammatory complications.

Data from scRNA-seq can reveal variability not recognized by phenotype-based methods and as such, scRNA-seq can provide insights into the heterogeneity of mechanisms and pathways underlying an individual’s cellular phenotype (Papalexi and Satija, 2018; See et al., 2018). By combining scRNA-seq with high dimensional immune profiling techniques we can attain detailed insights into an individual’s immune cell composition and link this to altered pathways and cellular function using multimodal analysis (Stoeckius et al., 2017). Alternatively, transcriptional analogs, like Cellular Indexing of Transcriptomes and Epitopes by Sequencing (CITE)-seq, which allows transcriptional evaluation of protein levels *via* nucleotide-tagged antibodies can be applied (Stoeckius et al., 2017). These techniques can be used simultaneously on a single cell (e.g., combining CITE-seq and RNA-seq) and analysis of the CITE-seq data can provide flow cytometry analogous cell subset evaluation, followed by transcriptional analysis within those identified cell subsets.

### 3.4 Epigenetics

Epigenetics focuses on quantifying the mechanisms by which gene expression patterns are altered without changing the nucleotides that make up the genome. The types of epigenetic modifications can be subdivided into DNA methylation, histone modifications by methylation, acetylation or phosphorylation and chromatin remodeling by various chromatin remodeling complexes. Epigenetic regulation is central to cellular health, can be altered by environmental influences and can be heritable (Hasin et al., 2017; Martínez-Cano et al., 2019). Techniques used to investigate how epigenetic modifications alter gene transcription, include chromatin Immunoprecipitation (ChIP)-sequencing, which is used to analyze how transcription factors (or proteins more generally) interact with DNA. For ChIP seq the interaction of selected proteins of interest with the DNA can be analyzed. Broader information is provided by Assay for Transposase-Accessible Chromatin with high-throughput sequencing (ATAC-seq) by assessing for variation in chromatin accessibility across the entire genome.

Epigenetic changes can play an important role in development of immune related diseases such as autoimmunity (Hewagama and Richardson, 2009; Meda et al., 2011; Brown and Wedderburn, 2015). Additionally, there are IEI caused by variants genes encoding for epigenetic regulators further stressing the importance of epigenetics in immune health. Examples of this include Immunodeficiency-Centromeric Instability-Facial Anomalies Syndrome 1(ICF1) caused by mutations in the DNA *de novo* methyltransferase 3B (DNMT3B) or Kabuki Syndrome caused by variants in KMT2D the gene encoding the histone methyltransferase Lysine-K-Methyltransferase 2D (MLL2) (Campos-Sanchez et al., 2019; Martínez-Cano et al., 2019). As epigenetic modifications can be acquired, they have been suggested to be involved in the development of secondary immunodeficiency (e.g., immune decline with aging or through malnutrition) (Martínez-Cano et al., 2019). Furthermore, epigenetic modifications have been suggested to contribute to the phenotypic variability found in patients with IEI (e.g., variability between monogenetic twins with Wiskott Aldrich Syndrome) (Buchbinder et al., 2011) and might influence disease development for individuals with disease causing variants of low penetrance (Campos-Sanchez et al., 2019). Furthermore, epigenetic modifications induced by the protein thymocyte selection-associated high mobility group box protein (HMG-box) chromatin associated protein (TOX) have been shown to lead to T cell exhaustion (Khan et al., 2019). Overall, assessing for epigenetic modification in addition to other phenotypic and functional assays can provide mechanistic insights into immune cell dysfunction. As above, ATAC-seq can be used simultaneously on a single cell (e.g., combining ATAC-seq, CITE-seq and RNA-seq) and analysis of the CITE-seq data can provide flow cytometry analogous cell subset evaluation, followed by transcriptional and epigenetic analysis within those identified cell subsets (Swanson et al., 2021).

### 3.5 Immune Cell and Serum Metabolic Analysis

The metabolic state of an organism, as well as of individual cells (including immune cells) changes based on a variety of conditions. Immune cells alter their metabolic state upon activation and when undergoing differentiation (and their baseline metabolic state impacts the way they respond to activation and differentiation stimuli). While immune cells alter their metabolic state to adequately respond to infections, altered T cell metabolism at baseline has also been observed in diseases such as obesity, autoimmunity, and cancer (Bantug et al., 2018). To study these processes, we use various techniques to both assess the metabolic function of immune cells (e.g., mitochondrial function and cellular metabolomics) and quantify humoral factors (e.g., serum metabolites).

Assessing mitochondrial function can be accomplished by quantifying and perturbing the metabolic capacities of immune cells. This can be done in bulk immune cells (or selected cell subsets) using techniques like Seahorse (Agilent). This technique has been used in IEIs, including Activated PI3K delta syndrome (APDS) and CD46 deficiency, since changes in T cell metabolism have been noted in both diseases (Lucas et al., 2014; Kolev et al., 2015). A major disadvantage of Seahorse is that it requires relatively large cell counts and can only be conducted on bulk cells. Emerging technologies such as Single-Cell ENergetic metabolism by profiling Translation inHibition” (SCENITH) could in the future allow us to combine readouts of cell metabolism on a single cell level with high-dimensional immune cell profiling (Argüello, 2020; Lopes et al., 2021). In this method, protein synthesis, as the most energetically intense cellular process, is measured in a given cell by quantifying the integration of puromycin, which is an antibiotic that incorporates itself into newly translated proteins and halts chain elongation. Puromycin can then be labelled by a fluorescent-tagged monoclonal antibody and can be used in combination with other antibodies and measured by flow cytometry. This reduces the cell counts needed and allows for the evaluation of mitochondrial function within immune cell subsets. SCENITH can thus allow us to link cellular phenotypes to metabolic alterations as a functional readout that could be therapeutically targeted. This technique remains distant from clinical use at this point, though insights gained by these strategies may inform clinical practice in the future.

Metabolomics, in contrast, encompasses the analysis of the various molecules produced by cellular metabolism by techniques such as liquid chromatography-mass spectrometry (LC-MS/MS). Substantial changes in the metabolic profile can arise in various disease states such as obesity, asthma, or cancer and these changes can induce immune cell dysfunction (Bantug et al., 2018; Wang et al., 2019; Hay and Henrickson, 2021; Porsche et al., 2021). Serum or cellular metabolomic profiles are rarely assessed in IEI, even though it is known that metabolites in the serum can be altered by inflammation and influence immune cell function, for example short-chain fatty acids can induce the differentiation of T cells into either effector or regulatory T cells (Park et al., 2015; Amelia, 2020; Hay and Henrickson, 2021). While an individual’s serum metabolomic profile might be used as phenotypic assessment (or as a source of biomarkers), it can also provide important mechanistic insights (e.g., metabolites such as acetate have been shown to directly influence immune cell function (Balmer et al., 2016; Hay and Henrickson, 2021)). Beyond the insight that can be gained by evaluating the metabolites bathing cells, cellular metabolomics can facilitate the evaluation of biochemical processes within immune cells. Better understanding the relationship between metabolites, cellular phenotype, function, and metabolism can thus not only provide important mechanistic information, but also potentially suggest novel therapeutic approaches (Lopes et al., 2021; Scharping et al., 2021).

### 3.6 Serum and Cell Lysate-Based Assays

Measurements of protein levels have long been part of the clinical and research-based evaluation of patients as they can provide a highly accurate representation of the active biological processes in a cell. While techniques such as Western Blots, enzyme-linked immunoassays ELISAs are commonly used, they only provide insights into the selected proteins. While there are various technologies that allow a broader, shotgun assessment of proteins their application is still limited largely by cost. Generally, methods such as ELISA or Western Blots are therefore still preferred for the targeted evaluation of protein expression.

Technologies such allowing for multiplex protein analysis (e.g., Olink. Luminex, Quanterix), allow for a broader, albeit defined, assessment of protein levels. They require only limited sample volumes and provide quick readouts and can thus be efficiently integrated into clinical workflows, though they are expensive. The technology that allows for the broadest assessment of protein levels is mass spectrometry. *Via* immunoprecipitation the technique can be utilized to analyze protein interactions and *via* enrichment techniques, such as those applied in phosphoproteomics, can allow for the quantification of phosphorylated proteins within a cell (Aebersold and Mann, 2003; Jünger and Aebersold, 2014). While these tools are extremely powerful, they still require significant amounts of protein, which may be beyond what is feasible from primary cells from a patient and is why cell lines are often required. Therefore, they are currently not applied in the clinics and are only rarely used to research IEI patients. Whether proteomics will manage to become more accessible remains to be seen, but some groups consider it to be a potential promising screening method for various IEI at birth (Collins et al., 2020). Regardless, methods of protein quantification will remain paramount in disease assessment, especially in combination with other techniques.

### 3.7 Evaluating the Microbiome

The study of the microbiome includes the diverse community of all microbes present in a specified microenvironment, such as the gut or the skin on a single patient or across a group of patients. Recent work regarding the central role of the microbiome in the development and function of immune cells in both healthy participants and those with various diseases makes its importance clear (Duffy, 2018; Javdan et al., 2020). In the intestine, the interplay between the microbiota and the immune system are central to the development and continuation of a healthy immune system (Castagnoli et al., 2021). While dysbiosis can result in immune disfunction, the immune system can also influence microbial composition (Jostins et al., 2012; Uhlig, 2013; Castagnoli et al., 2021). It is therefore not surprising that intestinal dysbiosis can be found in various IEI (Castagnoli et al., 2021). This can be assessed using metagenomic sequencing or microbial flow cytometry (Catanzaro et al., 2019).

While dysbiosis on its own is an interesting observation in IEI patients, recent studies are expanding our understanding of how alterations in the microbiome can alter immune cell function and contribute to disease progression (Chioma et al., 2021). One area of research focuses on how metabolites produced by the microbiome are influential contributors to disease progression (Haase et al., 2018; Kim, 2018). Additionally, an individual’s microbiome can contribute to their therapeutic response as it can influence the drug metabolism (Javdan et al., 2020; Balaich et al., 2021). As such the microbiome is an important contributor to individual immune cell function, clinical phenotype and therapeutic response that can be assessed in concert with other methods of immune cell evaluation.

### 3.8 Bespoke Functional Analysis

While a phenotype, such as alterations in T-cell activation markers, can suggest or imply a functional impact, assays that allow selective immune cell perturbation and functional evaluation are central to confirm suspected immune cell dysfunction. Almost all the above methods can in theory be used as readouts in part depending on the perturbation applied or cell types in question. As functional assays are usually quite specific, they are rarely conducted outside of research setting and are often only used in concert with extensive phenotypic evaluations. Furthermore, they may require large sample volumes and are therefore frequently conducted on cell lines, which can be done by genetically modifying primary cells or cell lines to express the patient variant or expanding primary cells. Cell lines specifically are useful tools in the functional evaluation of a mutated protein as often in IEI samples are rare and limited. Cell lines allow functional validation independent of patient samples. They can also remove the interpatient variability and be useful in confirming a variant to be disease causing (Casanova et al., 2014a). While there are several methods of generating cell lines, the most exciting potential comes from utilizing CRISPR to modify primary cells to match human genetic variants in IEI (Ralf, 1979).

In a research setting, functional assays (ranging from hypothesis-generating single cell transcriptional and epigenetic evaluation to bespoke assays designed for one gene or even the variant of interest) are often used to validate VUS or uncover novel disease mechanisms of known IEI. Overall, integration of functional tests into diagnostic workups are currently rare and require vigilant evaluation as well as expertise of the diagnostic laboratory. When they are optimized for future clinical use, they can be fundamentally useful in diagnosis or therapeutic selection. Currently most functional tests are still time consuming and need to be planned and conducted on a case-by-case basis. For a patient, requiring the evaluation of multiple VUS in several genes for example, finding and establishing functional assays for just one VUS in one gene already entails extensive clinical and research evaluation. We therefore need a strategy as a field to permit full evaluation of genetic testing, arguably most feasible by evaluating immune function more broadly.

Herein lies the potential of multimodal immune functional evaluation to revolutionize the diagnostic workup of IEI (Cols et al., 2016; Hsieh and Hernandez, 2016; Richardson et al., 2018). Based on key dysregulated pathways the most effective functional tests can be identified. By in turn integrating these as part of detailed phenotyping algorithms initial functional results can be attained quickly. The most informative and effective functional assays will need to be evaluated over time but establishing these techniques more broadly could circumvent the need to implement and validate an assay disease by disease. By focusing on alterations in key immune signaling pathways, rather than each VUS, we can build a more flexible and efficient diagnostic and therapeutic selection system in the future. This can and should be informed by genetic diagnosis where available but could be orthogonal.

## 4 MULTIMODAL FUNCTIONAL ANALYSIS

Although all the above-mentioned techniques are powerful modalities in and of themselves, they can only be used to their full potential when effectively combined. Currently this usually happens in research settings, here when optimally used in concert novel mechanistic insights can be attained, that not only deepen our understanding of the diseases in question but have broader implications in our understanding of basic immunological processes (Aguet, 2020; Katharina, 2022). While in some cases these results can have clinical implications for the patients studied, we are still far from their broad, standardized usage in daily clinical life. It is important to note that the primary aim of such research is not to inform clinical decisions. We can use it though, to better understand disease processes and select pathways to develop and optimize clinical testing for. This is important, as we currently do not know which techniques yield the greatest insight in which scenarios nor how to combine these techniques efficiently. In addition, both the cost, time and expertise required to perform these assays means that most will never be relevant clinically. Nonetheless, the insight we gain from these techniques can inform the selection of a subset of targeted tests to design and optimize for clinical use.

**FIGURE 5 |:**
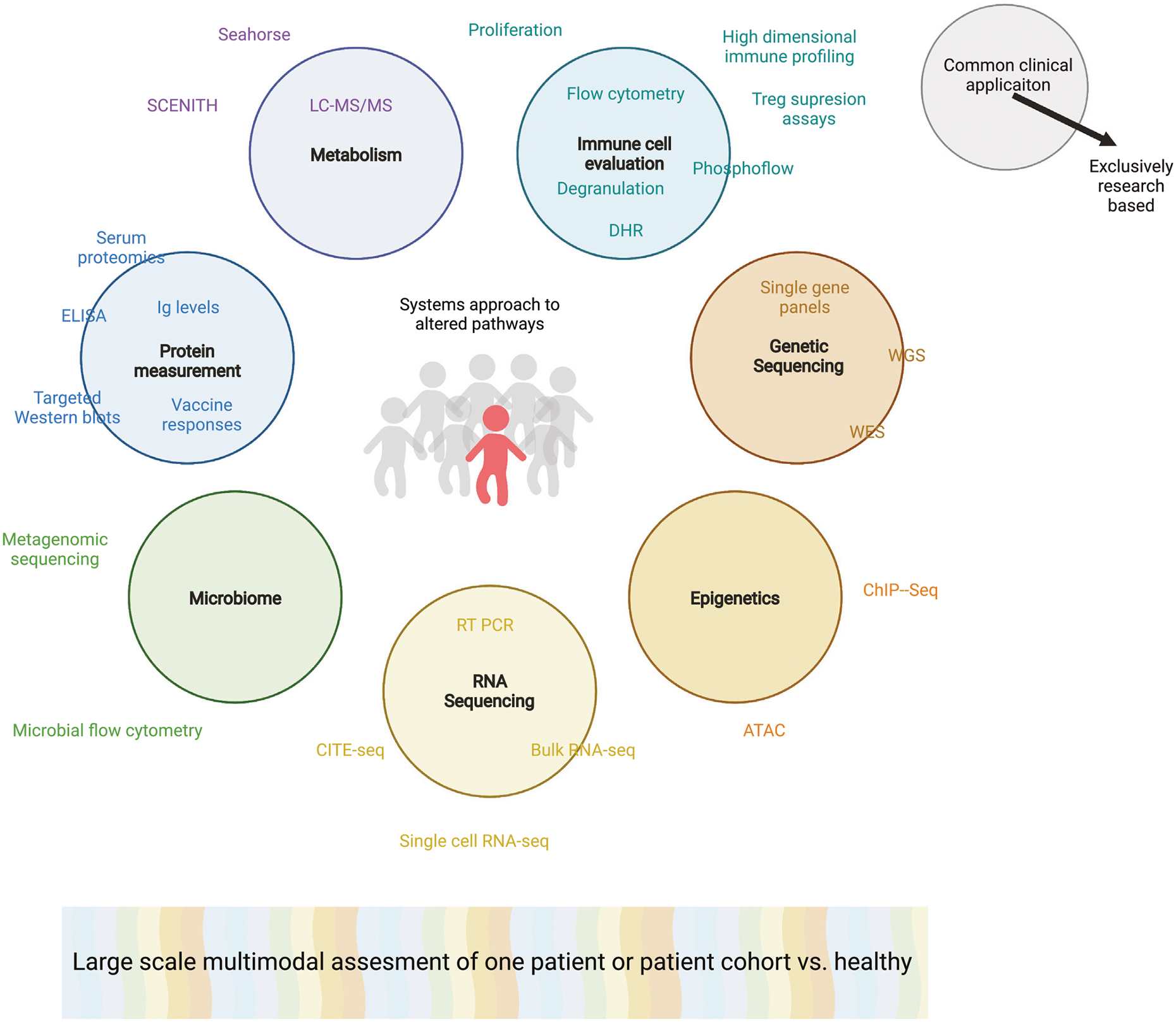
Multimodal analysis of a proband or complex patient or rare patients. Colored circles indicating various overarching systems biology techniques used to evaluate a patient’s immune cell phenotype or function. Corresponding specific techniques that may be used to attain this information are associated with each circle. Techniques within the circle are already commonplace as clinical diagnostic procedures while words outside of the circles are solely used on a research basis. The grey humans are healthy individuals while the red patient has a suspected PID. Broad multimodal analysis is depicted as a colorful spectra, highlighting the need to merge data across modalities. ATAC: Assay for Transposase-Accessible Chromatin with high-throughput sequencing; ChIP-seq: Chromatin Immunoprecipitation Sequencing; CITE-seq: Cellular Indexing of Transcriptomes and Epitopes by Sequencing; DHR: Dihydrorhodamine; ELISA: enzyme-linked immunoassays; Ig: Immunoglobulins; LC-MS/MS: Liquid chromatography-mass spectrometry; RT-PCR: Reverse Transcriptase Polymer Chain Reaction; SCENITH: Single-Cell Energetic metabolism by profiling Translation inhibition.

For the time being we are still learning from the first efforts to effectively combine these techniques and jointly analyze the high dimensionality of the data attained (Katharina, 2022; Swanson et al., 2021; Scharping et al., 2021; Aguet, 2020; Jha et al., 2015; Glass et al., 2020; Bernardes et al., 2020; Sachs et al., 1979; Jones et al., 2021). There are groups that are combining transcriptomics with genetics to identify rare genetic variants with pathogenic relevance (Aguet, 2020). While other groups have combined transcriptomic and metabolomic analysis to differentiate the mechanisms leading macrophage polarization (Jha et al., 2015). Using similar approaches, we as a field must initially focus on applying and optimizing the multimodal analysis of individual patients or patient cohorts (Figure 5). In order to effectively integrate data across modalities will require the thoughtful selection and combination of modalities and careful experimental planning including power calculations, ensuring that the conclusions drawn from this type of data analysis are reliable (Graw, 2021). This in concert with the ongoing research into healthy immune cell variation will hopefully allow us to optimize analysis algorithms that can identify the key dysregulated pathways that lead to an individual’s immune cell phenotype and underlie their defined dysfunctional phenotype.

Only when we have gained experience and data broadly using these various techniques on patient cohorts, can we recognize what modalities are most effective at understanding underlying altered pathways that can improve diagnosis and therapeutics. The goal is that doing “everything for a few” now in well characterized IEI patients and healthy controls, will hopefully allow us to identify what “few techniques to use for all” can be used in the future. By including in our analysis of our multimodal clinical data an individual’s clinical phenotype, we will hopefully learn to recognize patterns that can be applied to diagnose patients more effectively. The goal would be that for each individual patient we can use optimally selected techniques (that might not be high dimensional techniques) that in concert lead to a diagnosis. This final diagnosis might not be defined in all cases by a genetic variant but instead, especially in PIRD, by the dysfunctional pathways underlying the patient’s disease. For this to be of clinical relevance, as part of this research, we will need to evaluate the significance of these identified pathways, by applying targeted treatment strategies with the goal of normalizing immune cell function using *in vitro* assays before returning to the patients to improve therapy. In the future, these insights would ideally be codified to allow for identification of pathways to logically target in a specific clinical scenario with functional readouts.

## 5 ACCESS TO DIAGNOSTICS

One obvious issue hindering the broader adoption of a functional diagnosis for IEI patients based on multimodal high-dimensional data analysis includes the expense of these techniques and the degree of expertise required. Currently, many of these techniques they are only available in resource-rich institutions in wealthy countries. Nonetheless, the community of IEI researchers and clinicians alike collectively recognize that our current diagnostic and therapeutic algorithms need to be expanded. Currently, genetics are useful for some patients more than others; similarly clinical and immune cell phenotyping is helpful in some cases. Multimodal immune profiling in contrast is less constricted as it combines multiple techniques. When optimized, it promises to allow us to be more effective in diagnosing IEI and recognizing potential targeted treatment strategies. By additionally building on diverse databases of healthy individuals its application can broadly benefit our patients (Figure 4).

**FIGURE 6 |:**
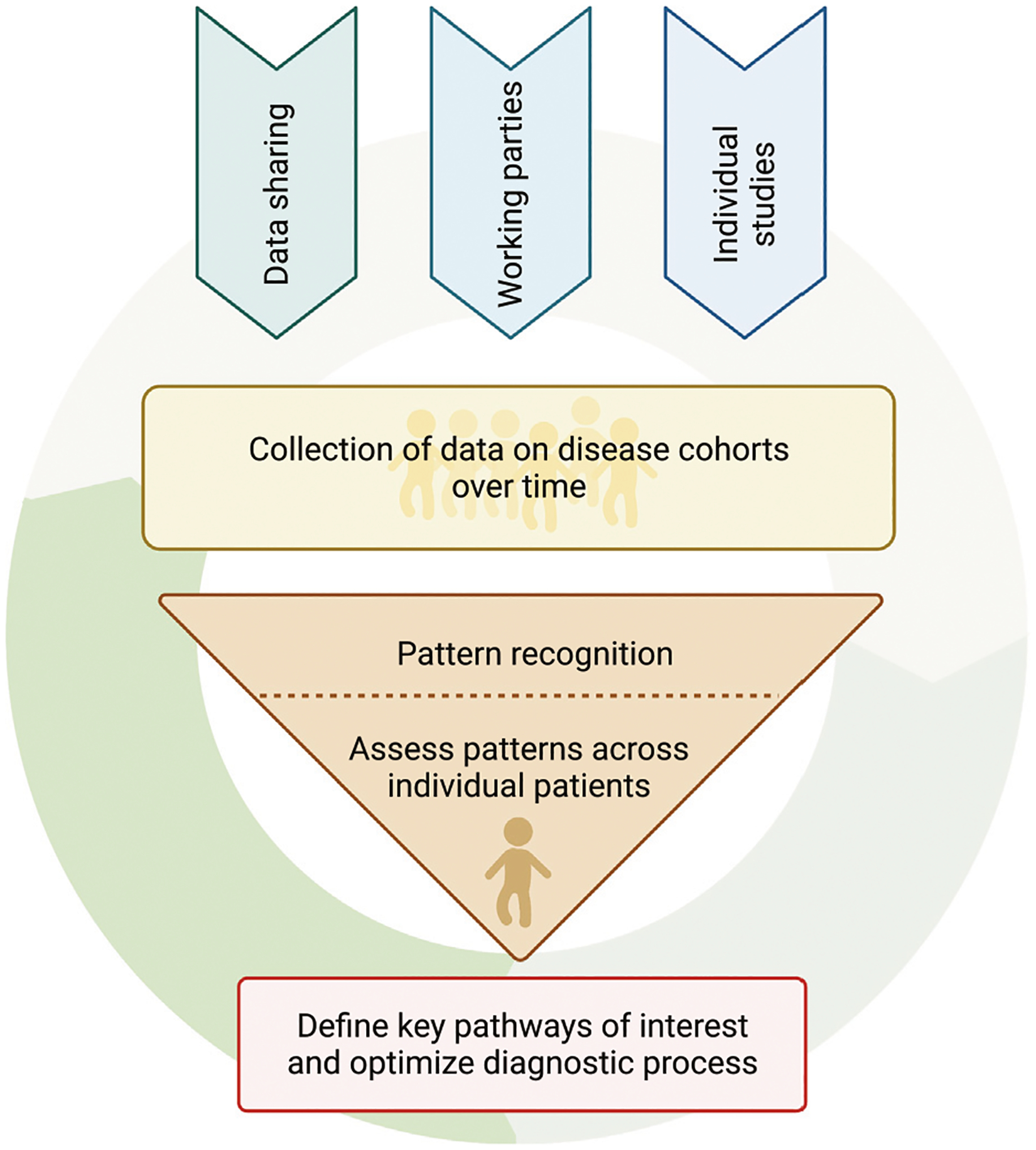
The pathway towards optimizing diagnostic algorithms for functional diagnosis of patients. The arrows above the circle depict the various means by which multimodal data can be obtained. The broad and coordinated collection of multimodal research data and analysis together with clinical metadata from individual patients can be evaluated to recognize specific functional alterations. These functional alterations and underlying altered pathways can then inform further specific data collection in future patients without diagnoses or groups of patients with known diagnoses and thus continue the cycle of data collection, data analysis and improved patient diagnosis.

Thus, to serve IEI patients worldwide, and to partner with our colleagues caring for these patients, it is our responsibility to use this deep multimodal research approach to foster identification and optimization of informed functional assays that are useful, actionable, and accessible (Figure 6). For long term equity, the investment in academia and intensified basic research is necessary to continue our advances. We at these academic institutions are in turn accountable to support research endeavors of colleagues and collaborators via sharing of knowledge and data. Furthermore, we need to foster the clinical endeavors to adopt these evidence-based techniques. Only by interdisciplinary, critical evaluation and discussion can we carefully select and establish resource-efficient modalities and educate our colleagues on their optimal application.

## 6 TREATING PATIENTS

### 6.1 Symptomatic Therapies

Before we focus on targeted therapies for IEI, we will highlight the symptomatic therapies that have effectively been used in IEI patients for decades. There are few IEI where antimicrobials, be they antibiotics, antifungals or antivirals, have not been central in disease management. Similarly, to this day, steroids and immune suppressants are effective treatment options for inflammation, autoimmunity and lymphoproliferation. Finally, replacement of immunoglobulins or infusions of blood products, such as erythrocytes and platelets, are often central to the management of IEI patients even before diagnosis. Often these symptomatic therapies alone, or in combination, are used for the management up to or long past their diagnosis. Our goal is to provide our patients with treatments that remove the need for these symptomatic therapies, although we currently do not always achieve this.

### 6.2 Hematopoietic Stem Cell Transplantation

Of course, symptomatic and targeted therapies are not the only treatments currently applied in IEI. Hematopoietic Stem Cell Transplantation (HSCT) is one of the mainstays in treating IEI, as historically, for many of the initially described IEI, this was the only potentially curative treatment option (Ochs and Hitzig, 2012). There are still IEI such as SCID, where HSCT is generally the optimal treatment choice. For these diseases quick diagnosis and allocation are central. We will not go into further detail on how multimodal functional assessment of patients can contribute to allocating patients for HSCT. We are still learning in PIRDs whether, how and when to best apply HSCT, therefor other techniques will surely remain fundamental to the treatment of these disorders. While we will next expand on targeted therapies, we will not go into further detail on other therapies such as gene therapy, that are being evaluated for select IEI.

### 6.3 Targeted Therapies

#### 6.3.1 Targeted to the Mutated Gene

Increased access to NGS has brought the promise of recognizing which signaling pathway(s) are defective in IEI patients, allowing for bespoke targeted therapies for the rare patients with IEIs based on the affected gene (Delmonte et al., 2019). The expanding clinical application of small molecule drugs and biologics, targeting specific signaling pathways markedly contributed to this expectation (Notarangelo et al., 2020). Currently, targeted therapies are generally directly up- or downstream of the mutated protein (Forbes, 2017; Forbes et al., 2018). There have been remarkable advances for patients using this strategy where applicable, though there have also been limitations for subsets of patients with those disorders.

Some examples of targeted therapies that have advanced the treatment of IEI patients include JAK inhibitors for STAT3 and STAT1 GOF or Abatacept for Lipopolysaccharide-responsive beige-like anchor (LRBA) deficiency (Lo et al., 1979; Forbes et al., 2018). For many patients these therapies have been highly successful and by widely applying these therapies we have learnt much regarding the mechanisms underlying these selected diseases. As mentioned in our STAT1 GOF excerpt though, there are still also a substantial number of patients (40% of patients being unresponsive in one systematic review of the literature) who do not respond to JAK inhibitors (Zhang, 2021). Therefore, as a field we recognize that we now need to continue building on these promising initial results from targeted therapies by delving into the pathways and mechanisms underlying treatment “failures.”

There are several potential factors that can reduce patient responses. For one, not all variants in a gene affect the resulting protein’s action in the same way. Depending on what aspect of the protein’s function is most affected, this could lead to very different outcomes on a cellular level. For another, one genetic variant can impact several signaling pathways, as mutated proteins often participate in various signaling cascades. Considering the interconnected nature of intracellular signaling and cellular interactions, this leads to effects in numerous downstream pathways. While these direct effects on alternative pathways can provide potentials for treatment, there are also compensatory mechanisms that can lead to alterations in seemingly unrelated signaling pathways. In some cases, these alterations might, in the end, contribute more significantly to the resulting cellular or clinical phenotype and thus provide more effective treatment avenues. Finally, how these changes alter the function of a whole system, such as the immune system, will depend on several other factors, that contribute to the overall functioning and robustness of the system. This includes factors like the microbiome, epigenetic modifications, and an individual’s metabolic state. Overall, one variant can have incredibly broad effects various cellular systems, that are difficult to predict. Thus, treating the pathway the gene is located within might not be the only or most effective therapeutic option—and we may need to deploy multiple strategies in some patients. Therefore, we need start evaluating additional therapeutic approaches, other than this purely genetics-based approach.

#### 6.3.2 Targeted to the Altered Pathway/Function

Based on this knowledge it becomes clear that potentially adjunctive targeted treatment strategies may emerge when we pivot away from thinking of IEI patients as being purely defined by their genetic variant towards a focus on incorporating the measurement of impaired function with the goal of returning cells to their normal state. Using the multimodal functional evaluations described above, we can begin to identify and apply targeted personalized therapies based on identified dysfunctional pathways.

As one example of creative strategies based on measured differences in immune phenotype and function, there are case descriptions of STAT1 GOF patients developing progressive multifocal leukoencephalopathy (PML) associated with JC virus (Zerbe et al., 2016). In PML several reports have noted the increased expression of PD-1 and suggested this to potentially hinder the clearance of JC virus. Therefore PD-1 blockade has been applied in several PML patients with some, albeit not all, patients showing promising responses leading to JC virus clearence (Tan et al., 2012; Berger, 2019; Cortese et al., 2019; Rauer et al., 2019; Walter et al., 2019). Whether elevated PD-1 in STAT1 GOF will be more widely described and building on this whether PD-1 blockade might provide an adjunctive therapy for STAT1 GOF patients will remain to be seen in the future but provides an interesting example of an alternative therapeutic option.

Defining functional alterations and pathways to target therapeutically, allows us to monitor the responses and adapt accordingly more quickly. Furthermore, moving away from a one gene, one treatment hypothesis means that treatments can be combined based on the different altered pathways and discontinued if not successful. Additionally, using multimodal approaches to diagnosing patients expands the therapeutic avenues. There are already successful examples using treatments to alter a patient’s epigenetic landscape (Zheng et al., 2015) or microbiome (e.g., fecal transplant) based on clinical and microbial phenotype (Wu et al., 2021). As part of this process, we may find new ways of treating IEI patients, by using novel therapeutic approaches or using known drugs with beneficial effects on unexpected pathways. Finally, this treatment approach can be flexibility adapted to a patient’s needs. As an individual reacts to environmental influences, such as infections or metabolic changes therapies can be adapted accordingly. Overall, this means that patients with very different variants might benefit from the same therapy for overlapping aspects of their disease. Understanding, that immune cell dysfunction is not static, means that patients will require regular reevaluation and might progress or circle through different therapies throughout their life. While this therapeutic approach is neither simple nor curative, it may better fit the complexity and variability of a subset of IEI patients that do not respond to classic therapies or meet classic disease phenotypes.

## 7 DISCUSSION

### 7.1 Main Findings

In this review we have covered the current state of systems biology in IEI and explored how these techniques are currently applied in diagnosing IEI patients, and how that might be improved going forward. By providing a historical perspective we aimed to highlight why the classification of IEI has developed as it has. This includes an exploration of how genetic testing has arguably become one of (if not the) primary diagnostic tool for IEI and both the significant advantages and limitations of this strategy. We focused on the importance of defining healthy immune cell variance as a prerequisite for each individual technique to accurately measure immune dysfunction in patient samples. Furthermore, we have expanded on several techniques that are used in immune phenotyping IEI patients, as well as the importance of adjunctive functional testing. We make the point that systems biology can improve IEI diagnosis and therapeutic selections by improving our ability to undertake targeted assessment of key pathways and cell subsets. Based on these results we can move towards establishing key, standardized, informative tests (that might not be systems biology based) to provide a functional diagnosis and treatment algorithm for each patient in the future.

By identifying the key altered pathways impacting function in a given patient, a personalized therapeutic approach can then be applied when treating these patients. To make functional diagnostics widely accessible, key nodes in the diagnostic and therapeutic algorithm will need to be identified. To achieve this, multimodal data analysis broadly applied over several techniques will need to be applied to broad groups of IEI patients and healthy controls over time. This will lead to insights for future evaluation of individual patients with immune dysregulation (Figure 5). By correlating these results to the individual’s clinical phenotype and therapeutic outcome we can iteratively select only the optimal diagnostic modalities for an individual with a suspected IEI (Figure 6). This could provide a novel means to effectively diagnose and treat IEI patients quickly and effectively.

### 7.2 Results in Context of the Existing Literature

Recent advances in systems biology allow deep analysis of gene and protein expression on a single cell level and the wider application of these techniques is leading to a rapid expansion of the field of systems biology. Research groups are often expert in one or two techniques, therefore the most effective means of combining techniques may come through collaborative research networks (Figure 4). These networks can also coordinate the recruitment of rare patient groups, ideally in collaboration with patient advocacy groups that focus on those diseases. Examples of such efforts already underway include those by various national and international consortia, including the Primary Immune Treatment Consortium (PIDTC) and various European Society of Immunodeficiencies (ESID) Working Parties, that aim to collaboratively collect data and coordinate research efforts on IEI (Griffith et al., 2016; Seidel et al., 2019).

Other examples of concerted efforts to share high dimensional data are most evident in the study of the healthy heterogeneity of cellular populations, previously not fully appreciated (Aguet, 1979; Kim-Hellmuth et al., 1979; Taylor et al., 2019; Jiang et al., 2020; Dumitrascu et al., 2021). Building on this strategy, coordinated efforts are in parallel being made in fields such as oncology, infectiology, rheumatology and inborn errors of immunity. For example, the collection of multiparametric data over time is being used to better understand the dynamic mechanisms underlying malignancy (Rozenblatt-Rosen et al., 2020). Similarly, during the recent COVID-19 pandemic the “COVID-ome Explorer research portal,” among others, was created to foster the sharing of high-dimensional data and coordinate research efforts (Sullivan et al., 2021). This work on large datasets is allowing multimodal data analysis as a means of drawing mechanistic conclusions to be optimized.

### 7.3 Implications for Inborn Errors of Immunity Research

High dimensional approaches on individual IEI patients or small patient cohorts can be used to attain important mechanistic insights into the disease (Casanova et al., 2014a). These can provide novel insights into many basic immunological processes that can have broader implications for our understanding of immunological processes at a basic science level. Therefore, research optimizing the functional diagnosis of IEI patients also has important implications for basic research.

As an IEI translational research community we need to make concerted efforts to collect and share this data and the necessary multimodal data analysis strategies across the rare diseases in our field and then work towards collectively defining what specific modalities can be used to effectively attain functional diagnoses in our patients. Then, we will need to monitor whether this functional diagnosis and suggested targeted therapies are effective and will need to define what future research is needed to optimize diagnosis but also what basic research is needed to better understand the disease mechanisms at play (Figure 6). As novel technologies arise, as our methods of data analysis improve and as we better control for variability amongst healthy controls, we will most likely find new patterns of potential dysfunction to home in on and the optimal tools to identify and differentiate them. Throughout this process, collaborative efforts around sample and data sharing will continue to be central (Figure 4). While these strategies can be very powerful, they can be challenging to build and expensive to maintain. Therefore, many collaborative efforts remain on a small scale, between pairs or small groups of research groups. This arguably reduces efficiency and rapidity of potential progress.

### 7.4 Implications for Practice

By strengthening existing and building new robust and inclusive collaborative networks internationally we will continue to improve our ability to best serve our rare patients. As part of this strategy, we will need to address how to integrate the findings in healthy individuals into our diagnostic procedures. One interesting aspect arises when we consider the findings that have been reported, as part of the concerted efforts to characterize healthy heterogeneity and healthy immune responses. Defining when healthy immune adaptation to aging, hormonal changes, diet and environmental factors ends and where disease begins, is difficult. As part of this we might find that certain IEI patients show substantial phenotypic overlap with patients suffering from more common diseases (especially where the altered pathways are shared between the two, for example, chronic inflammation and IEI characterized by amplified cytokine signaling)—this may yield fundamental insights into common disease mechanisms. Depending on how we conduct our multimodal analysis, we might try to find the patterns that differentiate our patients from the adaptations we find in healthy controls. Most likely, interesting insights into the disease mechanisms of various inflammatory diseases can be gained by focusing on the mechanistic similarities between these disease, potentially suggesting alternative therapeutic approaches for both.

Optimally, these all these concerted research efforts will provide clinicians with clear evidence based diagnostic pipelines that lead them to a functional diagnosis and therapeutic targets. Having identified key pathways that yield diagnostic and therapeutic insight, it will be necessary to develop and optimize clinical tests to measure these pathways in ways that can be used to drive clinical care. These diagnostic tests should at best be fast, targeted, and effective. Of note, though out of scope of this review, while establishing such pipelines can hopefully contribute to reducing the diagnostic delay of IEI, it does not inherently address the separate, broader issue of access to diagnostics throughout the world and should not remove focus from these endeavors. Providing patients with functional diagnoses based on mechanisms of immune dysregulation would ideally also inform the choice of therapeutic strategy by clinicians. As several pathways might contribute to dysfunction, a patient may receive several treatments or one therapy that affects multiple pathways. Further evaluation can be used to monitor therapeutic responses; thus, patients might discontinue or change therapies depending on whether their dysfunctional phenotype can be reversed. Here, once more, an iterative process will be needed to recognize what therapies work for which functional phenotype, in the context of a genetic diagnosis, when present.

## 8 CONCLUSION

Systems biology holds much promise of revolutionizing how we diagnose and treat IEI patients. For us to fully capitalize on its potential, we need to expand how we diagnose and treat IEI patients, continuing to build on the remarkable insights from the genetic revolution in IEI. Collaborative efforts will be needed to acquire extensive levels of data on selected IEI patients across the many rare disorders in our field. Only then can we use the multimodal data acquired to design more limited, evidence-based diagnostic testing and data analysis for our patients. Optimally these reduced diagnostic pipelines will be broadly accessible and effective in functionally diagnosing patients and suggesting treatment strategies. Thus, optimal application of systems immunology has the potential to decrease the diagnostic delay plaguing the field of IEI and increase access to effective functional diagnostics. This in turn can inform the selection of targeted therapies, providing patients with personalized precision medicine from diagnosis to therapy.

## QUICK GUIDE TO COMMON TERMS USED IN SYSTEMS BIOLOGY.

### Systems biology:

A field aimed at understanding complex biological systems. To this end, various high dimensional techniques are used to assess genes, molecules, proteins etc. of a biological system. What all techniques have in common is that methods of mathematical modeling and computational analysis are required to optimally explore the data.

### High dimensional data:

Data collected from methods that allow us to assess for more parameters than the number of samples. This makes data analysis complex and difficult with classical statistical methods.

### Multimodal analysis:

Combining results from different methodologies for one combined analysis. In systems biology this can mean combining multiple large and highly complex datasets.

### Data nodes:

Complex, connected data can be structured for analysis as a linked list or as a data-tree. Nodes are the connected points within these trees. A node can contain data or can represent a gene, protein etc.

### Data edges:

The lines connecting data nodes.

## DISEASE EXAMPLE: STAT1 GOF.

STAT1 GOF was initially described in 2011 in patients with chronic mucocutaneous candidiasis (CMC) (Liu et al., 2011; van de Veerdonk et al., 2011). Since this initial publication, over 600 patients have been reported in over 100 original publications. This reflects the extensive research into STAT1 GOF pathophysiology and makes it an optimal disease example to explore how the various techniques mentioned above are currently being applied to IEI research and where the current challenges lie. We will use this disease to exemplify how broader systems biology approaches could be applied to better diagnose and treat STAT1 GOF, as a representative IEI case study.

### Clinical variability of STAT1 GOF:

Defining the clinical phenotype of STAT1 GOF patients is challenging, due to the substantial clinical variation. Patients can be difficult to distinguish from a variety of other PIRDs. For example, while CMC is common, STAT1 GOF patients can also develop various other severe infections and be severely lymphopenic, making several combined immunodeficiency (CID) genes equally likely candidates upon initial workup (Eren Akarcan et al., 2017; Hartono et al., 2018; Al Shehri et al., 2019). A further laboratory phenotype that is extremely common in STAT1 GOF patients is the low Th17 cell count (Liu et al., 2011; Zhang, 2021), thus IL17 cytokine or receptor mutations and STAT3 LOF are common differential diagnoses in these patients (Eren Akarcan et al., 2017; Egri et al., 2021). Moreover, although STAT1 GOF is not considered to be a classic Type I Interferonopathy (Rodero and Crow, 2016; Bousfiha et al., 2020), there can be substantial clinical overlap (Smyth et al., 2018; Stellacci et al., 2019; Okada et al., 2020). Classic type I interferonopathies are a group of Mendelian disorders in which enhanced interferon (IFN) signaling is present. Patients are genetically and clinically heterogenous, but symptoms such skin vasculopathy and central nervous system involvement are considered to be pathognomonic. STAT1 GOF patients can present with overlapping symptoms, such as vessel calcification (Smyth et al., 2018; Stellacci et al., 2019) or systemic lupus erythematosus (Tirosh et al., 2019). On a biochemical level, STAT1 GOF mutations have been linked to increased downstream gamma-activating sequence (GAS) activity and a corresponding increase in the transcription of ISGs in various cell types (Liu et al., 2011; Sampaio et al., 2013; Soltész, 2013; Uzel, 2013; Kumar et al., 2014; Mizoguchi et al., 2014; Hirahara et al., 2015; Dotta et al., 2016; Hartono et al., 2018; Sampaio et al., 2018; Henrickson et al., 2019; Stellacci et al., 2019; Acker et al., 2020; Erdős et al., 2020). This overall makes STAT1 GOF an important differential diagnosis in patients with a phenotype suggestive of an interferonopathy.

Immune dysregulation is also very common in STAT1 GOF patients, making it difficult to discern from various other PIRDs. In some STAT1 GOF patients, autoimmunity can be the presenting phenotype, with presentations similar to IPEX (Immune dysregulation, polyendocrinopathy, enteropathy, X-linked). STAT1 GOF patients have accordingly been found in cohorts of IPEX-like patients without variants in FOXP3 (Uzel, 2013; Lee et al., 2020). Other cohorts in which STAT1 GOF patients have been found include common variable immunodeficiency (CVID) (Kobbe et al., 2016). Lymphoproliferation, as another sign of immune dysregulation, has been described STAT1 GOF patients, making STAT1 GOF a differential diagnosis for patients with concern for Autoimmune Lymphoproliferative Syndrome (ALPS) (Teachey and Bride, 2017; Henrickson et al., 2019). Similarly, STAT1 GOF can also underlie lymphoproliferative malignancies such as Hodgkin’s lymphoma, as well as other malignancies (Koo et al., 2017; Henrickson et al., 2019). Finally, HLH is a severe, life-threatening, hyperinflammatory pathophysiological process that has been described in multiple clinical scenarios, including some STAT1 GOF patients (Liu et al., 2011; Faitelson, 2014; Eng et al., 2020), thus making STAT1 GOF a potential, albeit rare, differential diagnosis in patients presenting with symptoms of HLH (Tabellini et al., 2017; Vargas-Hernández, 2018).

This phenotypic variability in STAT1 GOF patients is representative of that found in several other IEI and PIRDs. While this can make genetic diagnosis of this patients difficult it also raises substantial mechanistic questions regarding how this heterogeneity arises.

### Genetics in STAT1 GOF:

STAT1 GOF can be extremely difficult to recognize based on clinical presentation and laboratory parameters, but the use of genetics is also complex in this diagnosis. An “atypically” presenting STAT1 GOF patient could easily be missed if targeted sequencing does not include STAT1 and even if a STAT1 GOF mutation is correctly suspected, WES can still miss intronic variants (Hosking et al., 2020). While advances in NGS can address these issues, many of the issues with genetic sequencing discussed in the chapters above will hold true for STAT1 GOF as well. For example, somatic mutations that might be missed by NGS have been described in STAT1 GOF patients (Lee et al., 2020). Finally, it is difficult to predict the effect of a novel variant in STAT1 on the protein function. While in theory the clinical variation between patients with a loss of function (LOF) and GOF variant should help interpret genetic data, there is a surprising level of phenotypic overlap between STAT1 GOF and LOF (Kataoka et al., 2016; Toubiana et al., 2016; Pedraza-Sánchez, 2017; Olbrich and Freeman, 2018; Al Shehri et al., 2019; Zhang, 2021). Therefore, functional testing in adjunct to genetic testing is paramount in diagnosing these patients.

### Immune cell phenotyping:

STAT1 GOF serves as an illustration of how immune cell phenotyping is currently applied in diagnosing and managing IEI patients. Based on their clinical phenotype, STAT1 GOF patients are often initially referred for further immunological workup, which usually includes basic quantification of immune cells by flow cytometry. A review of the literature described low Th17 cells as the most common finding on flow cytometry in STAT1 GOF patients, while lymphopenia and hypogammaglobulinemia were less common (Zhang, 2021). Several reports have expanded on the mechanisms leading to Th17 deficiency, making the case that Th17 deficiency is the hallmark of STAT1 GOF patients (Liu et al., 2011; Mekki et al., 2014; Nielsen, 2015; Dhalla et al., 2016; Zhang et al., 2017; Hiller et al., 2018). While Th17 cell counts seem to be relatively sensitive in detecting STAT1 GOF, there are several other IEIs defined by low Th17 cells, including STAT3 LOF, thus reducing the specificity of this parameter (Puel, 1979). High dimensional immune profiling as a more sensitive means of immune cell phenotyping, has so far been published in three patients (Kaviany et al., 2021; Rosenberg et al., 2022). Here, it has uncovered an increased expression of T cell activation markers as a novel immune cell phenotype suggested to be potentially linked to immune dysfunction in these patients (Kaviany et al., 2021; Rosenberg et al., 2022).

Overall, while immune cell subset quantification alterations can be indicative of STAT1 GOF, it is not diagnostic and is currently used in combination with genetics and functional assays to diagnose patients. The most commonly used flow cytometry based functional assay for STAT1 GOF is the assessment of the timing and amplitude of STAT1 phosphorylation (pSTAT1) after interferon stimulation (Liu et al., 2011; Uzel, 2013; Giardino et al., 2016; Hartono et al., 2018; Olbrich and Freeman, 2018; Henrickson et al., 2019; Hosking et al., 2020).

### Transcriptional analysis in STAT1 GOF patients:

Alterations in STAT1 target genes by qPCR are commonly measured in primary cells and cell lines transfected to contain patient variants (Zheng et al., 2015; Zerbe et al., 2016; Meesilpavikkai et al., 2017; Bloomfield et al., 2018; Staab et al., 2020; Guisado Hernández, 2021). This is usually used to confirm increased STAT1 activity. RNA sequencing in turn has rarely been used, and when has mostly been applied to bulk cells to confirm an upregulation in known ISGs (Hirahara et al., 2015; Kayaoglu et al., 2021). scRNA-seq in STAT1 GOF patients has only been described in one paper where it was applied in concert with high dimensional immune cell profiling (Rosenberg et al., 2022).

### Epigenetics in STAT1 GOF patients:

Epigenetic alterations most likely play an important role in STAT1 GOF. While there are limited reports, it has been shown that in patient cells the epigenetic landscape is altered to promote the increased binding of STAT1 and thus increased activation of ISGs and decreased binding of STAT3 (Zheng et al., 2015; Kaleviste et al., 2019).

### Other means to measure protein levels:

In STAT1 GOF, methods to assess protein levels include ELISA for cytokine levels or Western blots for pSTAT1 levels.

### Metabolic assessment in STAT1 GOF:

One recent pre-print measures the levels of Glucose transporter 1 (Glut1) and carnitine palmitoyl transferase 1A (CPT1a) *via* CyTOF as a readout of cellular metabolic state in two STAT1 GOF patients (Kaviany et al., 2021).

### Functional assays in STAT1 GOF:

There are several functional assays commonly used to diagnose STAT1 GOF patients. While some of these can be conducted on primary cells, many groups bypass the need for primary cells by generating U3A or U3C cell lines, that do not express STAT1, transfected with a mutant and/or wild type plasmid (Soltész, 2013; Uzel, 2013; Henrickson et al., 2019; Lee et al., 2020; Staab et al., 2020). An alternative common method to increase availability of cells includes using immortalized patient derived cells, such as EBV transformed lymphoblastoid cell lines (LCLs) (Liu et al., 2011; Kumar et al., 2014; Pedraza-Sánchez, 2017). STAT1 phosphorylation following interferon stimulation can be assessed in these cell lines by western blots. Furthermore, cell lines can also be used for a luciferase reporter assay. By additionally transfecting the cells with a GAS coupled reporter system, this test can be used to confirm increased transcriptional activity of STAT1 upon cytokine stimulation (Soltész, 2013; Kumar et al., 2014; Mizoguchi et al., 2014). Finally, cell lines can be used to compare the effects different variants have on intracellular protein localization or gene transcription (Giovannozzi et al., 2020; Giovannozzi et al., 2021).

### Multimodal analysis:

Based on the current literature, multimodal analysis conducted on STAT1 GOF patients usually includes genomics and relatively low-dimensional phenotypical immune cell assessment followed by specific functional assays. This is a relatively common example of how our current assessment of IEI patients is completed and so far, has not allowed us to fully capture the mechanisms underlying the phenotypic variability seen in these patients. The first high-dimensional datasets (e.g., ChIP-Seq, CyTOF and scRNAseq) on STAT1 GOF patients indicate that there are several potential mechanisms (e.g., epigenetic modification, increased T cell activation etc.) at play in these patients (Zheng et al., 2015; Kaleviste et al., 2019; Kaviany et al., 2021; Rosenberg et al., 2022). For the time being, more high-dimensional data with age matched controls will be required to explore whether these signatures are characteristic of all STAT1 GOF patients and whether pathways underlying these changes can be found. Building on this, more extensive multiparametric, multimodal analysis of high dimensional datasets assessing not only phenotype, but function will optimally uncover specific patterns differentiating STAT1 GOF patients. This grouping of genetically similar patients by functional phenotype would then constitute a functional diagnosis. These patterns, linked to clinical phenotype can then allow us to suggest what selected techniques are required to functionally diagnose a patient based on their clinical phenotype.

As a completely hypothetical example of how this could play out, in a patient harboring a STAT1 GOF mutation primarily presenting with autoimmunity the information attained from diagnostic high-dimensional immune cell assessment with integrated metabolomic evaluation might reveal the specific and informative functional alterations. In another STAT1 GOF patient with infectious susceptibility we might primarily learn from genetics and research based scRNA-seq. Over time we might find that in STAT1 GOF genetics is not always be most effective means of informing diagnosis and therapy. By attaining this information for clinically genotypically diverse patients, in time diagnostics should become more specific and thus overall reduced. Optimally, by efficiently recognizing the key pathways underlying each individual’s functional diagnosis implications for clinical management can be drawn.

### Therapy in STAT1 GOF:

Currently, the primary targeted treatment of STAT1 GOF are Janus kinase (JAK) inhibitors, targeting directly upstream of the altered protein (Forbes, 2017). While there have been impressive successes using this treatment for several patients, others show a less marked or absent responses or severe side effects (Acker et al., 2020; Zhang, 2021; Deyà-Martínez et al., 2022). This most likely arises due to other pathways dysregulated (e.g., compensating) in unresponsive patients. A functional diagnosis of these patients could thus reveal alternative or adjunctive treatment strategies based on these other pathways. For example, high dimensional immune cell profiling has revealed elevated PD-1 in some patients (Kaviany, 2021; Rosenberg et al., 2022). This might offer itself as an alternative therapeutic target (Zerbe et al., 2016; Kaviany, 2021; Rosenberg et al., 2022). In selected STAT1 GOF patients, where increased PD-1 expression is substantial and correlates with dysfunction, PD-1 blockade might be effective, while in others, with less PD-1 expression, Ruxolitinib might stay the treatment of choice. This would constitute an example of how treating a functional phenotype could complement our current treatment approaches. Additionally, targeting epigenetic modifications has shown promise as a therapeutic option (e.g., treatment of patient cells with the histone deacetylase (HDAC) inhibitors trichostatin A or ITF2357) (Zheng et al., 2015).

## Abbreviations:

ATAC: assay for transposase-accessible chromatin with high-throughput sequencing
ChIP-seq: chromatin immunoprecipitation sequencing
CITE-seq: cellular indexing of transcriptomes and epitopes by sequencing
CMV: cytomegalovirus
CVID: combined variable immunodeficiency
DHR: dihydrorhodamine
EBV: Epstein Barr virus
ELISA: enzyme-linked immunoassays
Ig: immunoglobulins
JAK: Janus kinase
LC-MS/MS: liquid chromatography-mass spectrometry
RT-PCR: reverse transcriptase polymer chain reaction
SCENITH: single-cell energetic metabolism by profiling translation inhibition
SCID: severe combined immunodeficiency
STAT: signal transducer and activator of transcription
